# Horizontally Transferred Genetic Elements in the Tsetse Fly Genome: An Alignment-Free Clustering Approach Using Batch Learning Self-Organising Map (BLSOM)

**DOI:** 10.1155/2016/3164624

**Published:** 2016-12-15

**Authors:** Ryo Nakao, Takashi Abe, Shunsuke Funayama, Chihiro Sugimoto

**Affiliations:** ^1^Unit of Risk Analysis and Management, Hokkaido University Research Center for Zoonosis Control, Kita 20, Nishi 10, Kita-ku, Sapporo, Hokkaido 001-0020, Japan; ^2^Laboratory of Parasitology, Department of Disease Control, Graduate School of Veterinary Medicine, Hokkaido University, Kita 18, Nishi 9, Kita-Ku, Sapporo, Hokkaido 060-0818, Japan; ^3^Graduate School of Science & Technology, Niigata University, No. 8050, Igarashi 2-no-cho, Nishi-ku, Niigata 950-2181, Japan; ^4^Division of Collaboration and Education, Hokkaido University Research Center for Zoonosis Control, Kita 20, Nishi 10, Kita-ku, Sapporo, Hokkaido 001-0020, Japan; ^5^Global Station for Zoonosis Control, Global Institution for Collaborative Research and Education (GI-CoRE), Hokkaido University, Kita 20, Nishi 10, Kita-ku, Sapporo, Hokkaido 001-0020, Japan; ^6^Department of Disease Control, School of Veterinary Medicine, University of Zambia, P.O. Box 32379, Lusaka, Zambia

## Abstract

Tsetse flies (*Glossina* spp.) are the primary vectors of trypanosomes, which can cause human and animal African trypanosomiasis in Sub-Saharan African countries. The objective of this study was to explore the genome of* Glossina morsitans morsitans* for evidence of horizontal gene transfer (HGT) from microorganisms. We employed an alignment-free clustering method, that is, batch learning self-organising map (BLSOM), in which sequence fragments are clustered based on the similarity of oligonucleotide frequencies independently of sequence homology. After an initial scan of HGT events using BLSOM, we identified 3.8% of the tsetse fly genome as HGT candidates. The predicted donors of these HGT candidates included known symbionts, such as* Wolbachia*, as well as bacteria that have not previously been associated with the tsetse fly. We detected HGT candidates from diverse bacteria such as* Bacillus* and Flavobacteria, suggesting a past association between these taxa. Functional annotation revealed that the HGT candidates encoded loci in various functional pathways, such as metabolic and antibiotic biosynthesis pathways. These findings provide a basis for understanding the coevolutionary history of the tsetse fly and its microbes and establish the effectiveness of BLSOM for the detection of HGT events.

## 1. Introduction

Tsetse flies (*Glossina* spp.) are the primary vectors of trypanosome parasites; they cause human African trypanosomiasis (or sleeping sickness) and animal African trypanosomiasis (or nagana) in Sub-Saharan African countries. The flies harbour three maternally transmitted endosymbionts,* Wigglesworthia glossinidia*,* Sodalis glossinidius,* and* Wolbachia pipientis*, which influence host physiology. For example,* Wigglesworthia* provides essential nutrients, such as vitamins, to the host [1, 2] and influences host immune maturation [3]. Although the precise role of* Sodalis* in the tsetse fly is not clear, it appears to influence various host properties, such as longevity and susceptibility to trypanosome infections [4–6]. In many arthropod species [7, 8],* Wolbachia* induces strong cytoplasmic incompatibility, which was also observed in the tsetse fly [9]. In addition to these common bacteria, a recent microbial population analysis using a deep-sequencing approach revealed other facultative microorganisms from diverse bacterial families in the guts of tsetse flies, though their relative abundances were very low compared to that of the symbiont* Wigglesworthia* [10].

In addition to the parasitism of bacterial organisms themselves, partial genome sequences of* Wolbachia* are incorporated into the tsetse fly genome. Initially, Doudoumis et al. reported the incorporation of short fragments of three* Wolbachia* genes (16S rDNA,* fbpA,* and* wsp*) in the genomes of laboratory and natural* Glossina morsitans morsitans *(*Gmm*) populations [11]. Subsequently, a whole-genome sequencing project revealed large insertions of the* Wolbachia* genome in the* Gmm* genome via horizontal gene transfer (HGT) events [12, 13]. These insertions were identified by extracting* Wolbachia*-specific sequences from whole-genome Sanger sequencing reads and pyrosequencing data based on nucleotide homology with the complete genome sequences of three* Wolbachia* strains (wMel, wRi and wBm) [13]. Fluorescent* in situ *hybridisation analyses further confirmed the presence of these insertions in* Gmm* on the two sex chromosomes (X and Y) and the supernumerary B-chromosome [12, 13].

HGT elements can be detected by two main methods: phylogeny-based and composition-based methods [14]. The first method relies on sequence alignments; HGT is identified when the position of a query sequence in a tree does not match that of a reference phylogeny. Although this approach is robust, the frequency of HGT events may be underestimated, especially when there is a lack of information on donor sequences [15]. The second method relies on nucleotide compositional features, such as G+C content, nucleotide frequencies, or codon usage [16–18], and theoretically does not require sequence homology. Batch learning self-organising map (BLSOM) is an alignment-free clustering method that generates a map independently of the order in which data are input via a learning process [19, 20]. This method enables the clustering of genomic sequence fragments based on the similarity of oligonucleotide frequencies, without any other taxonomical information; it has been successfully applied in genomic and metagenomic studies [20–22].

The objective of this study was to characterise HGT from microorganisms in the genome of* Gmm *using the alignment-free clustering method BLSOM. Using BLSOM, we detected a number of HGT candidates from diverse origins. In a comparison of the results for HGT from* Wolbachia* between methods, there was a high level of agreement between BLSOM and BLASTn, a homology-based approach. Based on functional annotation, these potential HGT elements encoded loci in various functional pathways.

## 2. Materials and Methods

### 2.1. Genome Sequences

The tsetse fly (*Gmm*) genome (Accession number CCAG010000000) and all prokaryotic sequences identified to the species level (*n* = 5,600) were obtained from GenBank (http://www.ncbi.nlm.nih.gov/Genbank/). When the number of undetermined nucleotides (Ns) exceeded 10% of the window size (5 kb), the sequence was omitted from the analysis. When the number of Ns was less than 10%, the oligonucleotide frequencies were normalised to the length without Ns and included in the analysis.

### 2.2. Batch Learning Self-Organising Map

G+C% is a fundamental value for the phylogenetic classification of microbial genomes, including viral genomes, but it cannot differentiate a wide variety of genomes. Oligonucleotide composition can distinguish species, even those with the same G+C%, because it varies substantially among genomes; accordingly, it is referred to as a “genome signature” [23]. Multivariate analyses, such as factor correspondence analysis and principal component analysis (PCA), are useful to investigate variation in gene sequences [24]. However, the clustering power of conventional multivariate analyses is inadequate when massive quantities of sequence data from a wide variety of genomes are analysed collectively. Kohonen's self-organising map (SOM) is a powerful tool for clustering and visualising high-dimensional data vectors on a two-dimensional plane [25, 26]. To handle codon and oligonucleotide composition as high-dimensional data vectors, we modified the conventional SOM to develop the BLSOM [19, 20], which is suitable for genome sequence analyses and high-performance parallel computing. The initial weight vectors were defined by PCA, instead of random values, based on the finding that PCA can classify gene sequences into groups of known biological categories. Weight vectors (**w**_*ij*_) were arranged in the two-dimensional lattice denoted by* i* (=0, 1,…,* I*−1) and* j* (=0, 1,…,* J*−1). Weight vectors (**w**_*ij*_) were set and updated as described previously [19, 27]. A BLSOM program suitable for PC cluster systems is available on our website (http://bioinfo.ie.niigata-u.ac.jp/?BLSOM).

### 2.3. Detection of HGT Candidates in the Tsetse Fly Genome and Prediction of Their Origins Using BLSOM

To identify HGT candidates in the tsetse fly genome derived from prokaryotes, two types of large-scale BLSOM were used, that is, Tsetse+Prokaryotes- and Genus-BLSOM, using all genome sequences deposited in DDBJ/ENA/GenBank. A Tsetse+Prokaryotes-BLSOM was constructed with a degenerate tetranucleotide composition for all 5 kb sequences derived from tsetse fly genome sequences of longer than 5 kb plus 5,600 identified prokaryotes for which at least 10 kb of sequence was available from DDBJ/ENA/GenBank. The degenerate tetranucleotide composition was the composition of degenerate sets in which a pair of complementary tetranucleotides was added (e.g., ATGC and GCAT). To obtain more detailed phylotype information for the prokaryotic sequences, Genus-BLSOM was constructed for each phylum derived from 5,600 identified prokaryotes.

**Figure 1:**
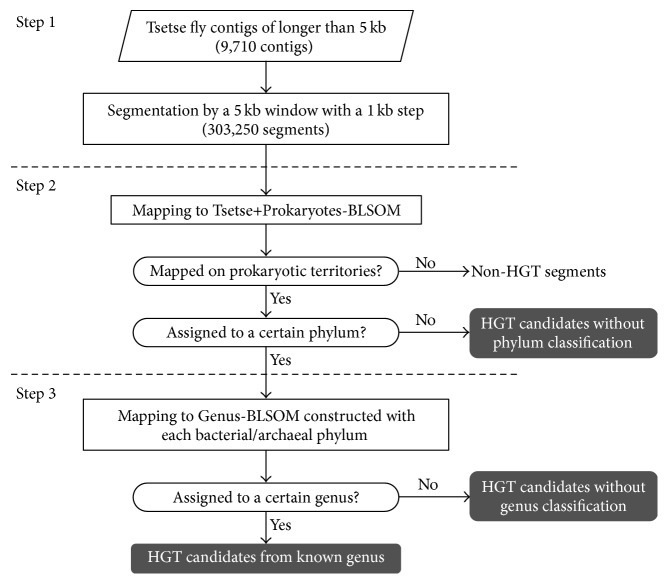
Workflow for data processing and BLSOM analysis.

For tsetse fly contigs of longer than 5 kb (9,710 contigs), a 5 kb window with a 1 kb step was used to obtain 303,250 segments (Figure 1, Step 1), which were mapped to Tsetse+Prokaryotes-BLSOM by identifying the lattice point with the minimum Euclidian distances in the multidimensional space (Figure 1, Step 2). For every lattice point at which tsetse fly genomic segments were mapped to prokaryotic territories, the most abundant phylum was identified, and the mapped tsetse fly genomic segments were tentatively assumed to belong to the phylum. Finally, when the most abundant phylum in more than 40% of the segments derived from a single tsetse fly contig was the same, the tsetse fly contig was assigned to this phylum by BLSOM. To identify the phylogenetic origin of the tsetse fly genomic segments that were mapped to the prokaryotic territories on Tsetse+Prokaryotes-BLSOM, they were successively mapped on Genus-BLSOM (Figure 1, Step 3). Similar stepwise mappings of tsetse fly genomic segments on BLSOMs constructed with sequences from more detailed phylogenetic categories (e.g., genera) were conducted.

### 2.4. Detection of HGT Candidates by BLASTn

To detect HGT candidates derived from* Wolbachia*, a local BLASTn search was conducted with the contig sequences of the tsetse fly that exceeded 5 kb against the NCBI nonredundant nucleotide database. When more than 1 kb of the sequence showed similarity with* Wolbachia* with top-hits and an* E*-value threshold of 1 × 10^−5^, the contigs were considered HGT candidates derived from* Wolbachia*.

### 2.5. Functional Classification of HGT Candidates

The sequence fragments with prokaryote origins were functionally annotated using KEGG (Kyoto Encyclopedia of Genes and Genomes) mapping [28] with the KAAS web server (http://www.genome.jp/tools/kaas/) [29]. KEGG Orthology (KO) assignments were obtained using the single-directional best-hit method. The organisms included in the analysis were as follows (based on IDs): hsa, dme, ath, sce, pfa, eco, sty, hin, pae, nme, hpy, rpr, mlo, bsu, sau, lla, spn, cac, mge, mtu, ctr, bbu, syn, aae, mja, afu, pho, and ape. The organisms in the database are listed on the KAAS web server (http://www.genome.jp/kaas-bin/kaas_org).

## 3. Results and Discussion

### 3.1. Detection of HGT Candidates Using BLSOM

**Figure 2:**
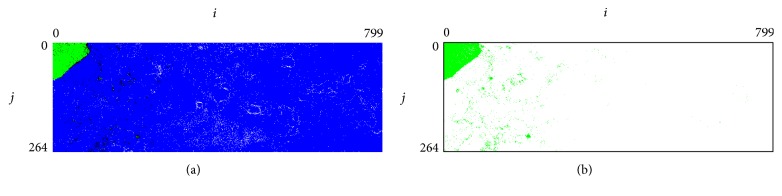
Tsetse+Prokaryotes-BLSOM. (a) BLSOM using the degenerate tetranucleotide set for the tsetse fly plus 5,600 identified prokaryotes. Lattice points that include the sequences from the tsetse fly are indicated in green, those that contain no genomic sequences are indicated in white, and those containing sequences from a prokaryote are indicated in blue. Lattice points that include both tsetse fly- and prokaryote-sequences are shown in black. (b) Distribution of tsetse fly genome sequences. Only green lattice points are shown.

Of 303,250 sequence segments obtained from the tsetse genome, we found that 11,524 sequences (3.8%) clustered with reads from prokaryotes and thus were HGT candidates according to Tsetse+Prokaryotes-BLSOM (Figure 2). These sequences were distributed across 2,960 different contigs, corresponding to 30.48% of all contigs. We assigned the most sequences to the phylum Firmicutes (*n* = 758), followed by the phyla Bacteroidetes (*n* = 370), Alphaproteobacteria (*n* = 90), and Gammaproteobacteria (*n* = 23) (Table 1). We did not assign 1,671 contigs to phyla owing in part to the presence of HGTs from multiple phyla within the same contig. It is also possible that these candidates were introduced by ancient HGT events, and their oligonucleotide compositions drifted over time [30], limiting the use of composition-based methods for classification.

**Figure 3:**
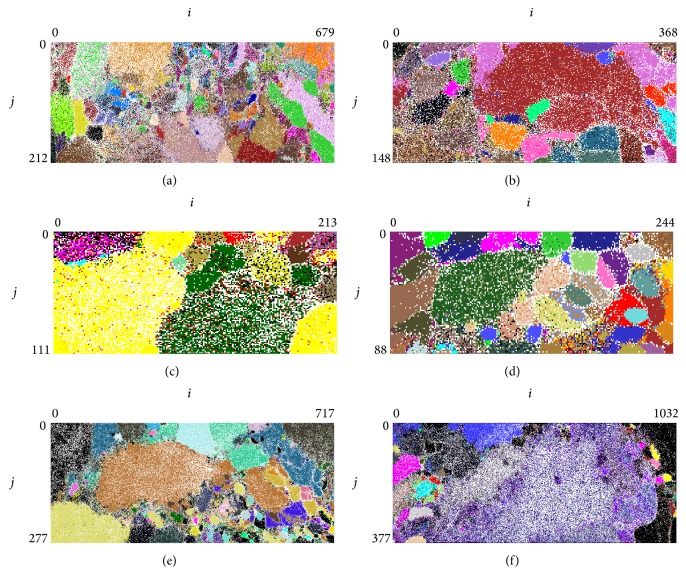
Genus-BLSOM. (a) Alphaproteobacteria. (b) Bacteroidetes. (c) Epsilonproteobacteria. (d) Euryarchaeota. (e) Firmicutes. (f) Gammaproteobacteria. Lattice points that include sequences from more than one genus are indicated in black, those including no sequences are indicated in white, and those including sequences from a single genus are indicated in individual color.

We performed further characterisation of donor sequences to the genus level using Genus-BLSOM (Figure 3). The results of four dominant phyla are summarised in Table 2. We assigned the most sequences to the genus* Bacillus* (*n* = 239). The second most highly represented origin was the class Flavobacteria (*n* = 187), which we were unable to classify to the genus level owing to coclustering with genome sequences that lacked genus information, followed by the genera* Staphylococcus* (*n* = 134),* Enterococcus* (*n* = 83),* Wolbachia* (*n* = 56),* Polaribacter* (*n* = 37), and* Listeria* (*n* = 31). The HGT candidates associated with the genera* Wigglesworthia* and* Sodalis*, which are common endosymbionts of the tsetse fly, were not detected using BLSOM. Most tsetse flies are heavily infected with* Wigglesworthia*; its abundance reaches over 99% in natural* Gmm* populations [10]. The lack of genome sequences associated with* Wigglesworthia* may support the high quality of tsetse fly genome sequences, since some genome data are contaminated by symbiont genomes, which can lead to the false-positive detection of HGT events [31]. This result also suggests that the symbiosis between* Wigglesworthia* and the tsetse fly was recent, as suggested by its genome features [32]. Nonetheless, we cannot exclude the possibility of bacterial genome contaminations in the tsetse fly genome since diverse bacteria exist in tsetse fly [10] and their sequences might not have been completely removed during the genome assembly process.

The high frequency of HGT candidates from the genus* Bacillus* suggests that there was a strong association between the tsetse fly and* Bacillus* in the past. Members of the genus* Bacillus* are ubiquitous in nature and have been isolated from diverse environments such as water, soil, plants, animals, and air [33]. Some species, such as* Bacillus thuringiensis*, have been well studied as agents of biological control of arthropods [34]. Kaaya and Darji infected several* Bacillus *species, including* B. thuringiensis*, to the adult* Gmm* and found that the mortality of* Gmm* was depending on the bacterial species [35], indicating that some* Bacillus* species may have infected* Gmm* persistently without adverse effect on the hosts and served as HGT donors. In fact, in a microbiota analysis of one tsetse fly species,* Glossina fuscipes fuscipes*, the bacteria belonging to the genus* Bacillus* were found dominant in a culture-dependent manner [36].

In contrast, there is no report on the relationship between the tsetse fly and Flavobacteria, which was identified as a second dominant donor of HGT candidates in this study (Table 2). Flavobacteria are symbionts in several arthropods [37–43], which indicates a high probability of the proliferation of this group of bacteria in arthropod hosts including tsetse fly. A comparative genome analysis of a flavobacterial symbiont (*Blattabacterium* strain Bge) in the omnivorous German cockroach (*Blattella germanica*) suggested that it plays roles in nutrient supply to the host, amino acid catabolism, and nitrogen excretion [41]. Flavobacterial symbionts in the ladybird (*Coleomegilla maculata*), and coccinellid beetle (*Adonia variegata*) induce male-killing [38, 39], in which male progeny in infected females die during embryogenesis. This phenomenon is widely recognised in other bacteria, such as* Wolbachia*,* Rickettsia*,* Arsenophonus*,* Spiroplasma,* and* Cardinium* [44]. Hurst et al. proposed that two male-killing symbionts cannot coexist at equilibrium in a single host species based on an observational study of the two-spot ladybird (*Adalia bipunctata*) infected with two symbionts,* Rickettsia* and* Spiroplasma* [39]. The presence of* Wolbachia* in the tsetse fly and HGT elements from* Wolbachia* in the tsetse fly genome [12, 13] may explain the absence of Flavobacteria in current tsetse fly populations.

### 3.2. HGT Candidates Derived from* Wolbachia*

We performed a BLASTn analysis to detect HGT candidates derived from* Wolbachia*. For 38 contigs, we detected sequence homology with* Wolbachia* sequences based on the criteria described earlier. Of these 38 contigs, we identified 36 as HGT candidates from* Wolbachia* using BLSOM, while we assigned the remaining two contigs to the genus* Rickettsia* using BLSOM. Accordingly, we observed a high level of agreement (36/38 = 94.7%) between the two approaches, indicating that the analytical sensitivity of BLSOM is at least comparable to that of BLASTn. These results also suggest that the genetic elements derived from* Wolbachia* were recently introduced to the tsetse fly genome. Based on a comparison between the BLSOM and BLASTn results, we observed 20 contigs that were only identified as HGT candidates using BLSOM. There may be as-yet unidentified* Wolbachia* strains, explaining the failure to identify HGT candidates based on sequence homology. In fact, for short regions (i.e., 338, 492, 497, and 497 bp) of four contigs identified as HGT candidates from* Wolbachia* only using BLSOM, we detected sequence homology with a* Wolbachia* endosymbiont of* Gmm* (Accession number AWUH01000121) with an* E*-value of <1 × 10^−5^.

Genetic elements related to* Wolbachia* that were presumably obtained by HGT have been detected in the genomes of multiple arthropod species, including the adzuki bean beetle (*Callosobruchus chinensis*) [45, 46], a fruit fly (*Drosophila ananassae*) [47], parasitoid wasps (*Nasonia* spp.) [47], the pea aphid (*Acyrthosiphon pisum*) [48], two mosquito species (*Aedes aegypti* and* Aedes mascarensis*) [49, 50], and the longicorn beetle (*Monochamus alternatus*) [51]. Some HGT events could be explained by nuclear-phage recombination, as proposed previously [49], but further studies are needed to determine the specific mechanisms of transfer. Since BLSOM can be used to detect* Wolbachia*-derived HGTs that exhibit low sequence similarity with known* Wolbachia* strains, it provides an alternative method with which to explore the mechanisms of HGT. The application of BLSOM to an increasing number of eukaryotic genomes will reveal the diversity and frequency of* Wolbachia*-derived HGTs in other arthropods, including vectors of medical and veterinary importance.

### 3.3. Functional Classification of HGT Candidates

We mapped all of the HGT candidates identified using Tsetse+Prokaryotes-BLSOM (*n* = 11,524) to the KEGG pathway. The KO included 317 biological pathways. The predicted pathways were mainly related to “Metabolic pathways” (193 molecules), “Biosynthesis of secondary metabolites” (75 molecules), and “Biosynthesis of antibiotics” (50 molecules). These results suggested that the HGT candidates have the potential to affect a large number of metabolic activities; however, further analyses are essential to demonstrate the active transcription of HGT-acquired genes using transcriptomics or gene-specific reverse transcription-PCR; such analyses can provide initial evidence for the functional importance of HGT-acquired genes [52]. In general, genes transferred to host genomes are pseudogenised via the acquisition of mutations, including insertions and deletions [46, 52, 53]. Unfortunately, since we could not employ RNA sequencing data into our analysis, it is not clear to what extent the detected HGT candidates have been pseudogenised. Nonetheless, active transcription of HGT-acquired genes has been detected in recipient hosts, such as a* Wolbachia*-derived gene in the* Aedes albopictus* C6/36 cell line [54]. Moreover, an increasing number of studies suggests that HGT-acquired genes facilitate the establishment of obligate mutualistic relationships between arthropods and their symbionts [55]. Analyses of the functional roles of HGT-acquired genes may improve our understanding of the complex interactions between the tsetse fly, microbes, and pathogens.

## 4. Conclusions

We investigated the use of BLSOM to detect HGT candidates in the tsetse fly genome. Using BLSOM, we successfully detected a number of HGT candidates from diverse bacterial origins. The HGT candidates represented 3.8% of the tsetse fly genome. The predicted donors of these HGT elements included* Wolbachia*, a well-known symbiont of the tsetse fly. In addition, using BLSOM, we identified HGT candidates from bacteria that have not previously been associated with the tsetse fly. We observed the HGT candidates from diverse bacteria such as* Bacillus* and Flavobacteria, suggesting a strong past association between these taxa. In a comparison between BLASTn and BLSOM results for the detection of HGT candidates from* Wolbachia*, the analytical sensitivity of BLSOM was at least comparable to that of the sequence homology-based approach. Furthermore, BLSOM can be used to detect HGT elements from organisms with low similarity with currently available sequences. These data obtained using BLSOM provide a basis for understanding the coevolutionary history of the tsetse fly and its microbes.

## Figures and Tables

**Table 1:** Origins of HGT candidates at the phylum level.

Phylum	Number of contigs
Actinobacteria	10
Alphaproteobacteria	90
Aquificae	2
Bacteroidetes	370
Betaproteobacteria	4
Crenarchaeota	1
Cyanobacteria	1
Epsilonproteobacteria	16
Euryarchaeota	12
Firmicutes	758
Fusobacteria	1
Gammaproteobacteria	23
Spirochetes	1
Unassigned	1,671

Total	2,960

Prediction was obtained using Tsetse+Prokaryotes-BLSOM.

**Table 2:** Origins of HGT candidates at the genus level.

Phylum	Genus^1^	Number of contigs
Alphaproteobacteria	*Anaplasma*	4
	*Bartonella*	8
	*Ehrlichia*	1
	*Neorickettsia*	8
	*Rickettsia*	5
	*Wolbachia*	56
	Unassigned	8

Bacteroidetes	*Bacteroides*	4
	*Cytophaga*	1
	*Dyadobacter*	1
	Flavobacteria^2^	187
	*Flavobacterium*	12
	*Kordia*	18
	*Leadbetterella*	3
	*Mucilaginibacter*	1
	*Paludibacter*	9
	*Pedobacter*	1
	*Polaribacter*	37
	*Prevotella*	10
	*Psychroflexus*	4
	*Spirosoma*	4
	Unassigned	78

Firmicutes	*Clostridium*	4
	*Bacillus*	239
	*Enterococcus*	83
	*Epulopiscium*	4
	*Erysipelothrix*	1
	*Geobacillus*	3
	*Lactobacillus*	4
	*Lactococcus*	1
	*Leuconostoc*	17
	*Listeria*	31
	*Lysinibacillus*	7
	*Oenococcus*	1
	*Paenibacillus*	1
	*Peptoniphilus*	1
	*Staphylococcus*	134
	*Streptococcus*	9
	*Thermoanaerobacter*	3
	*Turicibacter*	2
	*Veillonella*	3
	Unassigned	210

Gammaproteobacteria	*Acinetobacter*	4
	*Enterobacter*	6
	*Escherichia*	1
	*Haemophilus*	1
	*Shewanella*	1
	*Vibrio*	1
	*Xylella*	1
	Unassigned	8

^1^Prediction was obtained using Genus-BLSOM. ^2^Classification to the class level was obtained.
